# Acknowledgment to Reviewers of *Journal of Developmental Biology* in 2021

**DOI:** 10.3390/jdb10010008

**Published:** 2022-01-28

**Authors:** Journal of Developmental Biology Editorial Office

**Affiliations:** MDPI AG, St. Alban-Anlage 66, 4052 Basel, Switzerland

Rigorous peer-reviews are the basis of high-quality academic publishing. Thanks to the great efforts of our reviewers, *Journal of Developmental Biology* was able to maintain its standards for the high quality of its published papers. Thanks to the contribution of our reviewers, in 2021, the median time to first decision was 16 days, and the median time to publication was 44 days. The editors would like to extend their gratitude and recognition to the following reviewers for their precious time and dedication, regardless of whether the papers they reviewed were finally published:
Ackley, BrianMa, LiangAnanjeva, Natalia B.Maiorano, DomenicoAnderson, Matthew J.Mallela, Shamroop KumarAndrade, RaquelMandrioli, MauroArend, AndresMardešić-Brakus, SnježanaBalic, AnamariaMarrs, James A.Balnyte, IngridaMartinez, PedroBandyopadhyay, SushobhanaMaslen, Cheryl L.Baonza, AntonioMcDougall, AlexBarroso-González, JonathanMcGlinn, EdwinaBaschieri, FrancescoMeyer, Ralph G.Beck, CarolineMichiue, TatsuoBeedle, AaronMok, Gi Fay (Geoffrey)Beffa, Manuel MaríMoody, SallyBehm, ChristianMoshref, MaryamBelton, ChristianMulcahy, BenBénard, ClaireMüller, JulianeBlasi, FrancescoMunyanduki, HenryBogolyubova, IrinaO’Keefe, RaymondBrites, PedroOlsvik, Pål AsgeirBürglin, Thomas R.Osredkar, JoškoBury, StanisławPacherník, JiříCarnesecchi, JuliePapini, EmanueleCasacuberta, ElenaParker, Hugo J.Casares, FernandoPaz Montelongo, SorayaCaspe, Sergio GastónPeronnet, FrederiqueCastagnetti, StefaniaPetca, AidaCavagnetto, DavidePick, LeslieCerveny, KaraPleasure, SamuelChen, Jung-RenPlomp, Jaap JanChoe, Chong PyoProcino, AlfredoCovell, DavidRajendren, SubaCree, AlisonRos, MarianDariusz, SzarekSato, MasamitsuDomsch, KatrinScarpa, ElenaDonnay, IsabelleSchatten, GeraldEstella, CarlosSeiler, Magdalene J.Ethiraj, PurushothSen, RwikGil-Sanz, CristinaSeyedasli, NaisanaGiraldo, PilarShellard, AdamGoggolidou, ParaskeviShimada-Niwa, YukoGong, Siew-gingShimmi, OsamuHabibi, EhsanSion, GuyHenry, ClarissaSivakumar, SushamaHurley, James B.Steffen, DavidJaźwińska, AnnaSutherland, James D.Jeannotte, LucieTakiya, ShigeharuKalcheim, ChayaTurowski, GittaKasumyan, AlexanderVickaryous, Matthew K.Kidwai, ‪FahadVillalain, CeciliaKölbel, HeikeVonhoff, FernandoKostyuchenko, RomanWanner, NicolaKucharczyk, DariuszWartiovaara, KirmoKulakova, MilanaWaters, Samuel T.Layer, Paul G.Watt, KristinLemke, SteffenWilson-Rawls, JeanneLin, TaoWong, Yin PingLohmann, Ingrid


